# Patterns of Gray Matter Abnormalities in Idiopathic Generalized Epilepsy: A Meta-Analysis of Voxel-Based Morphology Studies

**DOI:** 10.1371/journal.pone.0169076

**Published:** 2017-01-06

**Authors:** Guo Bin, Tianfu Wang, Hongwu Zeng, Xiaoming He, Feng Li, Jian Zhang, Bingsheng Huang

**Affiliations:** 1 National-Regional Key Technology Engineering Laboratory for Medical Ultrasound, Guangdong Key Laboratory for Biomedical Measurements and Ultrasound Imaging, School of Biomedical Engineering, Health Science Center, Shenzhen University, Shenzhen, Guangdong, P.R, China; 2 Department of Radiology, Shenzhen Children’s Hospital, Shenzhen, Guangdong, P.R, China; 3 Department of Neurology, Xiangyang Central Hospital/Affiliated Hospital of Hubei University of Arts and Science, Xiangyang, Hubei, P.R, China; 4 Department of Radiology, Xiangyang Central Hospital/Affiliated Hospital of Hubei University of Arts and Science, Xiangyang, Hubei, P.R, China; 5 School of Medicine, Health Science Centre, Shenzhen University, Shenzhen, Guangdong, P.R, China; Chinese Academy of Sciences, CHINA

## Abstract

**Objective:**

We aimed to identify the consistent regions of gray matter volume (GMV) abnormalities in idiopathic generalized epilepsy (IGE), and to study the difference of GMV abnormalities among IGE subsyndromes by applying activation likelihood estimation (ALE) meta-analysis.

**Methods:**

A systematic review of VBM studies on GMV of patients with absence epilepsy (AE), juvenile myoclonic epilepsy (JME), IGE and controls indexed in PubMed and ScienceDirect from January 1999 to June 2016 was conducted. A total of 12 IGE studies, including 7 JME and 3 AE studies, were selected. Meta-analysis was performed on these studies by using the pooled and within-subtypes analysis (www.brainmap.org). Based on the above results, between-subtypes contrast analysis was carried out to detect the abnormal GMV regions common in and unique to each subtype as well.

**Results:**

IGE demonstrated significant GMV increase in right ventral lateral nucleus (VL) and right medial frontal gyrus, and significant GMV decrease in bilateral pulvinar. For JME, significant GMV increase was seen in right medial frontal gyrus, right anterior cingulate cortex (ACC), while significant GMV decrease was found in right pulvinar. In AE, the most significant GMV increase was found in right VL, and slight GMV reduction was seen in right medial dorsal nucleus, right subcallosal gyrus, left caudate and left precuneus. No overlapped and unique regions with significant GMV abnormalities were found between JME and AE.

**Significance:**

This meta-analysis demonstrated that thalamo-frontal network was a structure with significant GMV abnormality in IGE, and the IGE subsyndromes showed different GMV abnormal regions. These observations may provide instructions on the clinical diagnosis of IGE.

## Introduction

Idiopathic generalized epilepsy (IGE) is a group of epileptic disorders with high prevalence across all ages[1]. According to predominant clinical symptoms and age onset, IGE can be either childhood or juvenile absence epilepsy (AE), juvenile myoclonic seizures (JME), and generalized tonic–clonic seizures (GTCS) on awakening [1]. Some studies in patients with clinically homogeneous IGE pointed to distinct genetic abnormalities among IGE subsyndromes, supporting the existence of different subtypes[2,3]. Despite the clinical difference, sometimes the difference of EEG pattern among IGE subsyndromes were not always consistent[4], especially when several IGE subsydromes coexisted[5]. Blurred distinctions for IGE subsydromes also occurred in the measurement by magnetic resonance imaging (MRI). Conventional MRI was usually normal in patients with IGE, and it is often difficult to detect the subtle brain abnormalities, if exist, for IGE. Thus, the differentiation between IGE subsydromes have been very challenging.

Over the past years, quantitative evaluations based on the voxel-based morphology (VBM) have enhanced the sensitivity of MRI on brain abnormalities detection in IGE patients. The VBM studies in IGE suggested that VBM was sensitive in detecting the subtle structural alterations which cannot be measured by conventional MRI[6]. However, these findings from VBM studies were inconsistent among IGE studies. In IGE, one study by Tae et al found prefrontal lobe atrophy[7], whereas some other researchers reported prefrontal lobe hypertrophy[8,9]. In some studies no GMV changes were found[10,11], however in some other studies, GMV alterations were not only found in the thalamus and frontal lobe, but also in other brain regions such as the parietal lobe, insula, temporal lobe, cingulate gyrus[12,13].

Such limited generalizability and inconsistent results in VBM studies may be attributed to the small sample sizes and difference in data processing algorithm. To address this issue, meta-analysis has emerged as a systematic and comprehensive method used to derive a pooled estimate closest to the unknown common truth[14]. Particularly, meta-analysis based on activation likelihood estimation (ALE) has been designed and utilized effectively in some neurological diseases[15]. With ALE technique, every single VBM foci are described as Gaussian probability distributions representing their underlying spatial uncertainty[16]. These distributions are pooled in a voxel-wise fashion within and across a group of experiments to generate a corresponding whole-brain ALE-map. Each voxel within the ALE-map represent the probability of a specific experiment effect. These maps are then tested against a null distribution, with a user-defined statistical threshold to determine clusters of significant meta-analytic convergence.

To the best of our knowledge, no ALE meta-analysis of VBM studies in IGE has been carried out. In this study, by applying ALE analysis we aimed to identify the consistent regions of GMV abnormalities in IGE, JME and AE compared to the controls, and between-subtypes contrast analyses were then utilized to determine the significantly abnormal regions common or unique to JME and AE.

## Materials and Methods

### Literature search and inclusion

This study is performed based on the PRISMA guidelines, please see S1 Checklist. A comprehensive and systematic search was performed in PubMed and ScienceDirect Database (from January 1999 to June 2016) for VBM studies of IGE, by using the following keywords: “gray matter” AND (“epilepsy” OR “JME” OR “juvenile myoclonic epilepsy” OR “GTCS” OR “generalized tonic-clonic seizure” OR “AE” OR “childhood or juvenile absence epilepsy” OR “IGE” OR “idiopathic generalized epilepsy”) AND (“Voxel-based morphometry” OR “VBM” OR “Voxel-wise”). For the studies obtained, each was respectively scanned by a professor who specializes in neuroimaging and an experienced neurologist, to determine if it met the inclusion criteria. The references in the selected studies were also reviewed to identify the relevant papers.

The included studies should meet these criteria: (1) full text is accessible and published in English with peer review; (2) reporting a VBM comparison on gray matter volume between IGE patients and healthy controls; (3) reporting the stereotactic coordinates of significant GMV abnormalities across the whole brain; (4) corrected significance levels for multiple comparisons, or uncorrected levels with spatial extent thresholds were used.

A study was eliminated if (1) there was no healthy control group; (2) this study were not reported on Talairach or Montreal Neurological Institute (MNI) stereotactic coordination; (3) the data overlapped with other articles; (4) the reported changes were uncorrected and the spatial extent threshold was not reported; (5) the patients were infants or newborns; (6) the subject information was insufficient. The method used in the present study was according to the Meta-analysis of Observational Studies in Epidemiology (MOOSE) guidelines for observational studies[17].

### ALE meta-analysis

For comparing the GMV difference and seeking a consistent anatomical bias among IGE subsyndromes, we used BrainMap database (http://www.brainmap.org/), which is an online database of structural neuroimaging studies in the form of stereotactic(x,y,z) coordinates, including most of the published VBM studies (most papers included in our study can be found in this database). Thus, the above collected VBM studies, if included in this database, were firstly retrieved, and the associated information and coordinates were extracted from the Brainmap database using the tool Sleuth 2.3.6 (http://www.brainmap.org/). Experiments unavailable in the database were manually encoded using Scribe 2.3.1 (http://www.brainmap.org/). Coordinates in Talairach & Tournoux (T&T) space were converted to Montreal Neurological Institute (MNI-152) space by using the icbm2tal transform in ALE.

In this study, we performed pooled analysis to compare the difference of GMV abnormalities between IGE and controls, and within-subtypes analysis to compare the difference of GMV abnormalities between JME and controls, or AE and controls, by using cluster-level thresholding of ALE algorithm. When using cluster level inference, the simulated data are thresholded by using a “cluster-forming threshold”. GingerALE finds the contiguous volumes of clusters above the threshold, and then tracks the distribution of their volumes by using permutation threshold. False discovery rate (FDR)-corrected threshold inference was used to control the rate of false positive. In this study, pooled and within-subtypes analysis was performed by using a cluster level threshold of 0.05, a permutation threshold of 1000, and a FDR-corrected P value of less than 0.05. Notably, we did not perform meta-analysis for GTCS, since there were very few studies (1 for GMV increase, 2 for GMV decrease) about this subtype.

For comparing the difference of GMV abnormalities between JME and AE, we made a contrast analysis for these two subtypes. In this between-subtypes contrast analysis, GingerALE created simulated data by pooling the foci datasets and randomly dividing them into two new groups, which had the same size as the input data sets. For example, JME contained 20 foci, while AE consisted of 29 foci, then the pooled data of 49 foci were therefore randomly divided into two groups, one 20 and the other 29 foci. ALE values were calculated for each group, and then compared to the ALE values of the true data. After 1000 permutations, a null distribution was generated for the difference in ALE values between JME and AE. The true difference in ALE values was then tested against this null hypothesis at each voxel, generating a voxel-wise P-value image that was thresholded with a FDR <0.05 and a minimum cluster size of 100 mm^3^.

In addition, conjunction analysis was carried out for JME and AE to assess the common regions with GMV abnormalities. This was derived from the voxel-wise minimum value of the input ALE images. The resulting conjunction image reflects the statistically significant similarities between the JME and AE.

Mango software (http://ric.uthscsa.edu/mango) was employed to visualize the ALE results, which were overlaid on the MNI-152 brain template in MNI coordinate space.

## Results

**Fig 1:**
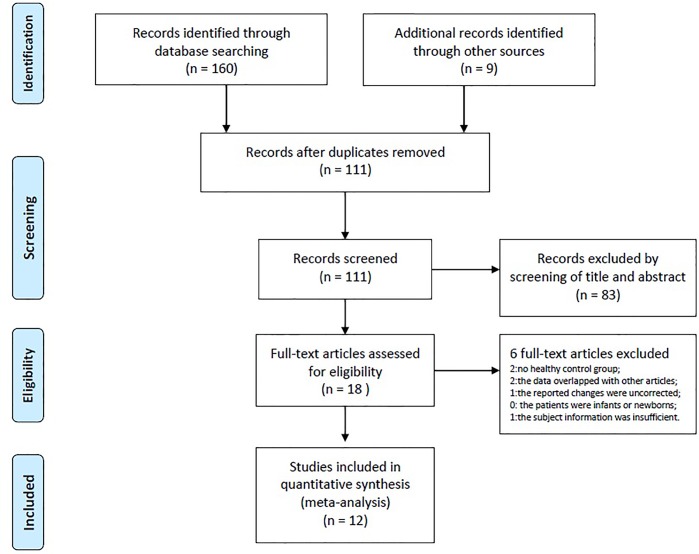
Flow diagram of studies included in the current review.

**Fig 2:**
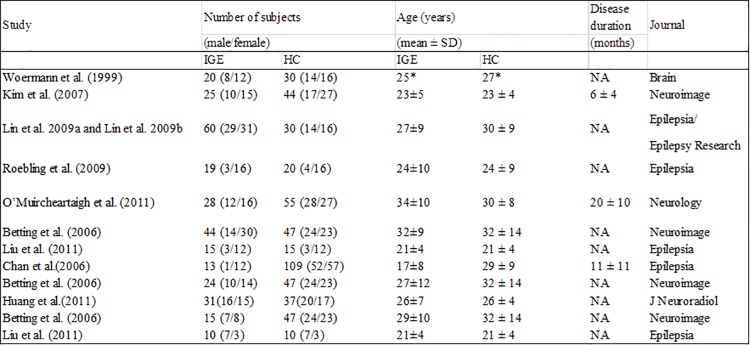
Summary of studies included in the ALE meta-analysis. ALE, activation likelihood estimation; IGE, idiopathic generalized epilepsy; VBM, voxel-based morphometry; HC, healthy controls; SD, standard deviation; NA, not available;* Median age.

**Fig 3:**
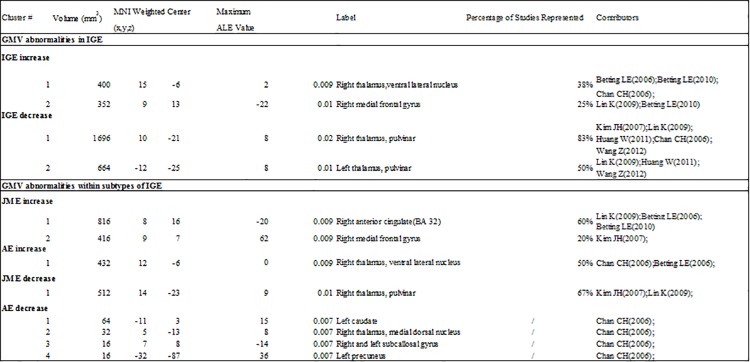
ALE results of gray matter volume comparison in IGE subsyndromes. ALE, activation likelihood estimation; IGE, idiopathic generalized epilepsy; JME, juvenile myoclonic epilepsy; AE, absence epilepsy; MNI, montreal neurological institute.

**Fig 4:**
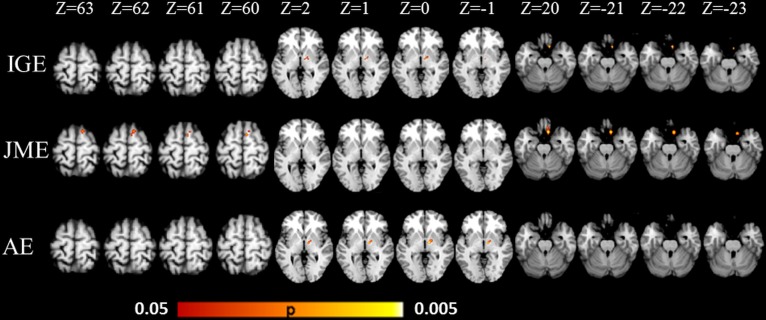
Results of ALE meta-analysis with GMV increase. Significant GMV increase was found in: for IGE, right ventral lateral nucleus and right medial frontal gyrus; for JME, right medial frontal gyrus and right anterior cingulate; and for AE, right ventral lateral nucleus. IGE, idiopathic generalized epilepsy; JME, juvenile myoclonic epilepsy; AE, absence epilepsy; ALE, activation likelihood estimation; GMV, gray matter volume.

**Fig 5:**
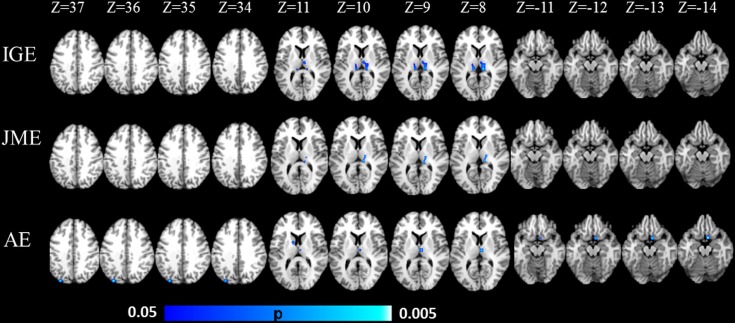
Results of ALE meta-analysis with GMV decrease. Significant GMV decrease was found in: for IGE, bilateral pulvinar; for JME, right pulvinar; and for AE, right medial dorsal nucleus, right subcallosal gyrus, left caudate and left precuneus. IGE, idiopathic generalized epilepsy; JME, juvenile myoclonic epilepsy; AE, absence epilepsy; ALE, activation likelihood estimation; GMV, gray matter volume.

The detailed information of the papers selection are summarized in Figs 1 and 2. The results about GMV increase or decrease in IGE subsyndromes compared to controls are detailed in Figs 3–5.

### GMV abnormalities in IGE

IGE demonstrated significant GMV increase in right ventral lateral nucleus (VL) and right medial frontal gyrus (Figs 3 and 4). Significant GMV reduction was found in bilateral pulvinar in IGE, and this was the only cluster exceeding 1000 mm^3^ (Figs 3 and 5).

### GMV abnormalities within subtypes of IGE

For JME, the most consistent regions with significant GMV increase was located in right medial frontal gyrus and right anterior cingulate cortex (ACC, Brodmann area 32), and these clusters presented in more than 80% of the studies about JME (Figs 3 and 4). The cluster with significant GMV decrease was found in right pulvinar (Figs 3 and 5).

The most significant GMV increase was found in right VL in AE, which was reported in 50% of the studies about AE (Figs 3 and 4). Slight GMV reduction was seen in right medial dorsal nucleus (MDN), right subcallosal gyrus (frontal cortex), left caudate and left precuneus (parietal cortex) (Figs 3 and 5).

### GMV abnormalities between subtypes of IGE

There were no regions with significant GMV abnormality shared by JME and AE. No regions unique to any of the subtypes were found either.

## Discussion

We in the present meta-analysis study summarized the GMV changes in IGE based on the previous VBM studies. We found increased GMV in VL and medial frontal gyrus, and decreased GMV in pulvinar bilaterally in IGE patients; increased GMV in medial frontal gyrus and anterior cingulate cortex, and decreased GMV in pulvinar in JME patients; increased GMV in VL and decreased GMV in MDN, prefrontal cortex, left caudate and parietal cortex in AE patients. However, there no specific and common abnormal regions between these two subtypes.

### GMV abnormalities in IGE

It was found that GMV increased in VL and decreased in pulvinar in IGE patients. Both VL and pulvinar are located ventral to thalamus. Generally, thalamus receives numerous inputs including sensory and motor signals from cerebral cortex, and then integrates and processes these signals before projecting them onto the cortex[18]. Largely because of this ability in regulating between cortical and subcortical structures, thalamus is of great importance in generalized seizure. In EEG-fMRI studies, it is often observed that the abnormal activity in thalamus starts before or continues till the end of the generalized seizure wave discharges (GSWD), thus, one may conclude that, within the connection of thalamocortical circuitry, thalamus is likely to play a key role in the initiation to the propagation of GSWD[6]. It is reported that thalamus is responsible for a variety of complex tasks such as sleep, attention and arousal state [19,20]. Relevant behavioral and neuroimaging studies have found that IGE patients show abnormal sleep architecture and arousal system, and worse attention [21], and have further proved that these disorders are closely related to the thalamus activities[22]. Thus, our findings about GMV abnormalities in VL and pulvinar may explain the underlying morphological mechanism of IGE with such disrupted functions.

In addition, patients with IGE showed GMV increase in medial fontal gyrus (MFG), which was the only cortical area with significant GMV abnormalities in this study. As a part of prefrontal cortex, MFG is one of the key brain regions that form the network particularly related to execution and cognition[23]. fMRI and behavioral studies have showed that patients with IGE get abnormal performances in the tests of executive and cognitive function[24]. Collectively, The GMV abnormalities within this structure might underlie these poor test performances of IGE patients.

Notably, as thalamus receives abundant afferent connections from prefrontal cortex, close associations exist between these two structures, and it appears very important in the cognition regulating[25]. By summarizing our findings in these two regions (thalamus and prefrontal cortex), and in combination with the previous findings that the thalamo-frontal connectivity showed significant abnormalities in IGE group[26], a conclusion may be drawn that these structural deficits in thalamo-frontal network may be important biomarkers of IGE.

### GMV abnormalities within subtypes of IGE

GMV decreased in right pulvinar and increased in medial frontal gyrus in JME group, and these findings generally overlapped with previous meta-analysis results[27]. There is a consistent viewpoint that the most important network in JME is the circuit between thalamus and medial prefrontal cortex[28], namely the thalamo-prefrontal circuit, which plays an important role in the regulation of various motor tasks. Evidences from fMRI studies suggested that JME patients showed degenerated cognitive and executive skills, and these poor performances were related to atypical activities within the thalamo-prefrontal circuit[29]. Thus, our results may provide morphological evidence for the underlying mechanism of these functional observations.

ACC (Brodmann Area 32) was another region with significant GMV alternation in JME. This area is associated with rational thinking process, and most notably active during the Stroop task [30]. Behavioral studies suggested that JME patients showed more errors in Stroop test [31,32], and atypical ACC was found to be partly correlated to these poor performance[33,34]. Collectively, the abnormalities of ACC reported in our study may be the potential structural explanation for these behavioral deficits.

GMV abnormalities extending from thalamus to frontal and parietal cortex were found in AE group. Recently, it was found that close association existed between frontal and parietal cortex[35], and this frontal-parietal connectivity and relevant thalamic structures were highly correlated with consciousness regulation[36]. AE has been considered as the most common seizure type with loss of consciousness, and the impaired consciousness in AE patients may be partly attributed to the disordered neural activities in frontal-parietal network [37]. Our results echoed this interpretation that abnormal frontal-parietal connectivity was involved in AE patients. Another region with GMV reduction in AE was caudate, which has been considered important in the cognitive regulation[38]. Evidence from fMRI studies suggested that atypical caudate was part of the salience network in AE, and this abnormality may cause damages on cognition and attention in AE patients[39]. Collectively, the abnormalities extending from subcortical to cortex observed in our study may help to clarify the underlying mechanism of AE from a structural perspective.

### GMV abnormalities between subtypes of IGE

In between-subtypes contrast analysis, there were no regions with significant GMV abnormality shared by JME and AE. This result is well supported by the findings from within-subtypes analysis, in which no overlap of regions with GMV abnormality was found between JME and AE (Fig 3).

However, we did not find any significant abnormal region unique to each subtype. The reason may partly exist in the small data sets. As mentioned before, only 7 JME experiments and 3 AE experiments were included in our study, thus we may not have enough statistical power to show the significant difference in between-subtypes contrast analysis with less than fifteen experiments in each data set (http://www.brainmap.org/). Actually, among these involved VBM studies, one has suggested that different patterns of GMV abnormalities existed between JME and AE[40]. In addition, many neuropathological and EEG studies showed that both AE and JME are focal seizure with distinct abnormal regions[41,42]. Collectively, it is believed that the GMV abnormalities are specific between JME and AE, but more samples are needed to verify this interpretation.

### Limitation

Our study may have several limitations. Firstly, we were unable to analyze the correlation between the disease duration and GMV abnormalities due to the limited VBM source data, hence a direct observation about the changes of GMV abnormalities with disease duration in patients cannot be depicted. Secondly, although significant statistical rigor was exercised in utilizing the revised ALE meta-analytic technique, our study was nevertheless based on the summarized stereotactic coordinates, rather than the raw imaging data, and these summarized statistical data may lead to inaccuracy in the final conclusion. Thirdly, the heterogeneity of the methodology, such as in data pre-processing, image modulation and template registration of these VBM studies, may potentially affect the accuracy of our results. Fourthly, in this study we excluded the studies published in languages other than English. Improvement in data management, data collection and data processing techniques would help to solve these limitations and to draw a more robust conclusion.

### Conclusion

This meta-analysis demonstrated that thalamo-frontal network was a structure with significant GMV abnormalities in IGE, and these GMV abnormalities were more located in unilateral thalamo-frontal network in JME group, while extended from thalamus to frontal and parietal cortex in AE group. However, it was incapable of revealing the significant overlapped or specific regions with GMV changes between JME and AE with the limited experiments involved. Future studies examining larger samples may better elucidate the difference between IGE subtypes and highlight their morphological distinctions, and may finally help to improve the management of IGE patients.

## Supporting Information

S1 Checklist PRISMA 2009 Checklist. (DOC)
